# Fast and Cost-Effective Mining of Microsatellite Markers Using NGS Technology: An Example of a Korean Water Deer *Hydropotes inermis argyropus*

**DOI:** 10.1371/journal.pone.0026933

**Published:** 2011-11-01

**Authors:** Jeong-Nam Yu, Changman Won, Jumin Jun, YoungWoon Lim, Myounghai Kwak

**Affiliations:** 1 National Institute of Biological Resources, Environmental Research Complex, Incheon, Korea; 2 Department of Life Sciences, Ewha Womans University, Seoul, Korea; Auburn University, United States of America

## Abstract

**Background:**

Microsatellites, a special class of repetitive DNA sequence, have become one of the most popular genetic markers for population/conservation genetic studies. However, its application to endangered species has been impeded by high development costs, a lack of available sequences, and technical difficulties. The water deer *Hydropotes inermis* is the sole existing endangered species of the subfamily Capreolinae. Although population genetics studies are urgently required for conservation management, no species-specific microsatellite marker has been reported.

**Methods:**

We adopted next-generation sequencing (NGS) to elucidate the microsatellite markers of Korean water deer and overcome these impediments on marker developments. We performed genotyping to determine the efficiency of this method as applied to population genetics.

**Results:**

We obtained 98 Mbp of nucleotide information from 260,467 sequence reads. A total of 20,101 di-/tri-nucleotide repeat motifs were identified; di-repeats were 5.9-fold more common than tri-repeats. [CA]_n_ and [AAC]_n_/[AAT]_n_ repeats were the most frequent di- and tri-repeats, respectively. Of the 17,206 di-repeats, 12,471 microsatellite primer pairs were derived. PCR amplification of 400 primer pairs yielded 106 amplicons and 79 polymorphic markers from 20 individual Korean water deer. Polymorphic rates of the 79 new microsatellites varied from 2 to 11 alleles per locus (*H_e_*: 0.050–0.880; *H_o_*: 0.000–1.000), while those of known microsatellite markers transferred from cattle to Chinese water deer ranged from 4 to 6 alleles per locus (*H_e_*: 0.279–0.714; *H_o_*: 0.300–0.400).

**Conclusions:**

Polymorphic microsatellite markers from Korean water deer were successfully identified using NGS without any prior sequence information and deposited into the public database. Thus, the methods described herein represent a rapid and low-cost way to investigate the population genetics of endangered/non-model species.

## Introduction

Microsatellite markers are appropriate for use in population studies because they are ubiquitous, co-dominant, economically feasible, and reproducible with high rates of polymorphism [1]–[3]. A microsatellite simple sequence repeat (SSR) is a stretch of DNA with a repeated mono-, di-, tri-, or tetra-nucleotide that shows a high level of length polymorphism due to insertion or deletion mutations of one or more nucleotides, generated mainly by slipped-strand mis-pairing during DNA replication [4],[5]. As a locus-specific marker, these are used in many fields, including behavior studies using relatedness, conservation genetics, population structure analyses, medical studies, and forensics. The advantages of using these markers include random distribution throughout the genome and only a small quantity of genetic material required for genotyping [5]–[7].

Currently, numerous microsatellite-development methods are available. Of these, the simple hybrid capture method is the most popular choice for non-model species because it does not require any prior sequence data. The repetitive sequences are captured using a biotinylated probe and selectively attached to magnetic beads. After several washing steps, the captured DNA fragments are eluted, amplified, and cloned to produce a clone library enriched for the target sequences. Following DNA sequencing of positive clones, PCR primers are synthesized and tested [8]. However, this conventional microsatellite-development method is inefficient and includes many technically difficult steps such as cloning and making/screening the enriched library [9]. Thus, this method is laborious, has a low success rate, and requires at least 1–3 months to develop microsatellites, even when experienced personnel are involved.

The most cost-effective microsatellite-development method is to use publicly available genetic/genomic information on the species of interest. The microsatellites derived from publicly available sequences can be divided into genomic and gene microsatellites [10]. Genomic microsatellites are located in non-coding sequences (introns or intergenic spaces). This type of microsatellite is usually derived from BAC (Bacterial Artificial Chromosome)/fosmid (end) sequences and RFLP (Restriction Fragments Length Polymorphism) clone sequences. Gene microsatellites were designed from coding sequences, mainly obtained from EST (Expressed Sequence Tag) analysis. Genomic microsatellites are useful for population studies because they are more polymorphic and selectively neutral than gene microsatellites [10], [11]. However, molecular sequence data from most wild species remains limited, particularly data from regions such as ribosomal DNA or EST sequences; these are less polymorphic than non-coding DNA regions at the population level.

The final method is using transferable microsatellites from very closely related species. For example, 14 of 88 microsatellite markers designed from the EST library of the zebra finch were successfully amplified and revealed polymorphism among at least five passerine species from five different genera [12]. Also, microsatellites designed from the cultivated sunflower *Helianthus annuus* EST database were successfully transferred to the closely related wild species *H. verticillatus* and *H. angustifolius* [13]. However, microsatellite primers are typically only transferable among very closely related species and do not address the problem of a lack of sequence information from non-model species. Microsatellite development using NGS (Next Generation Sequencing) could overcome such a lack of sequence information [14]–[16]; however, only a limited number of genotyping experiments have been performed to test this method [17], [18].

*Hydropotes inermis* Swinhoe, 1870, a small cervid, was recently placed close to the roe deer in the tribe Capreolini, sub-family Capreolinae, based on molecular phylogenetic study [19]. *H. inermis* contains two recognized subspecies, distinguished by their geographic distribution and body color: *H. inermis inermis* in China, and *H. inermis argyropus* in Korea [20]. Several decades ago, two parapatric subspecies were widely distributed in east China and the Korean peninsula but numbers have been drastically reduced by habitat loss and poaching [21], so it is classified as Vulnerable (VU) by the International Union for the Conservation of Nature (IUCN) [22]. Thus, conservation management and captive-breeding programs are now targeting Chinese water deer [23]. Despite the importance to taxonomy and conservation, few reports of genetic studies of this species at the population level have been published [24]–[26], and to our knowledge no water deer-specific microsatellite markers are known.

Therefore, we examined the efficacy of NGS-based microsatellite development using *H. inermis*, and confirmed the utility of the derived microsatellites by amplification of target sequences and genotyping of 20 Korean water deer. Compared to previously reported cross-species microsatellite markers, NGS-based microsatellite development was both rapid and cost-effective. Therefore, microsatellite development using NGS will accelerate detection of microsatellite markers in endangered/non-model species and thereby facilitate population genetics studies that will themselves contribute to the management and conservation of such species.

## Materials and Methods

### Sample preparation and NGS

Genomic DNA was isolated from frozen muscle tissues of 20 road-killed Korean water deer (found by researchers at the Ministry of Environment and sent to the National Institute of Biological Resources for species identification and longterm storage under Korean domestic laws related to wildlife conservation) using a DNeasy Blood and Tissue Kit (Qiagen, Germany). One specimen among 20 individuals was used to screen microsatellite fragments using NGS. After checking the quality of genomic DNA by resolution on a 1% agarose gel and spectrophotometry (Nanodrop, USA), approximately 10 µg genomic DNA was subjected to sequencing using a 1/4 plate of a Roche 454 GS-FLX titanium platform at NICEM (Seoul, South Korea). To assess the amplification success and polymorphism of the newly designed microsatellites, all 20 specimens were used for genotyping.

### Microsatellite discovery

We obtained ∼98 Mbp of sequence data (260,467 sequence reads) that contained 63 Mbp of assembled contigs and singletons for *H. inermis*. The sequence reads were assembled using the Newbler software package (Roche Diagnostics, 454 Life Science). Since sequence information was only used for primer design and their sequences were verified by Sanger-sequencing with designed primers, we assumed that its low coverage and low read depth did not hamper the entire experimental design for screening microsatellites. After sorting the assembled contigs and single reads according to size, those shorter than 100 bp were discarded because small fragments are not appropriate for PCR amplification and genotyping. All perfect di- and tri-nucleotide repeats (CA/AC/GT/TG, TA/AT, GC/CG, CT/TC/GA/AG, AAT/ATA/TAA/ATT/TTA/TAT, AGG/GGA/GAG/TCC/CCT/CTC, ACC/CAC/CCA/TGG/GTG/GGT, AGC/GCA/CAG/TCG/CGT/GTC, AAG/AGA/GAA/CTT/TTC/TCT, ATG/TGA/GAT/TAC/ACT/CTA, AAC/ACA/CAA/TTG/TGT/GTT, AGT/GTA/TAG/TCA/CAT/ATC, ACG/CGA/GAC/TGC/GCT/CTG, GGC/GCG/CGG/CCG/CGC/GCC) in a database of the remaining sequences were searched using the ‘ssr_finder.pl’ perl program [27]. The obtained di-repeats were sorted according to the number of repeats using the data filter command in Microsoft Excel. A pair of primers flanking each repeat were designed using the Primer3 software package (http://www-genome.wi.mit.edu/cgi-bin/primer/primer3_www.cgi) [28]. The optimal primer size was set at 22 bp (range 18–26), the optimal annealing temperature was set at 58°C (range 55–62°C), and the remaining parameters were left at the default settings.

### Verification of microsatellites

One hundred primer pairs from 20 Korean water deer with the largest number of di-repeats were selected for amplification. A 5′-M13 tail [29] was added to each forward primer to allow fluorescent labeling during amplification. Each 10 µl PCR reaction included 1 µl template DNA (30–50 ng), 0.5 units DNA polymerase (TAKARA, Japan), 1 µl 10× PCR buffer containing 25 mM MgCl_2_ (TAKARA, Japan), 0.8 µl dNTPs (20 mM), 0.25 µl forward and 1 µl reverse primers (16 µM each), and 1 µl fluorescently labeled M13 primer (16 µM; 6-FAM, VIC, PET and NED). Amplification conditions were as follows: 5 min pre-denaturation at 94°C followed by 30 cycles of 30 s at 94°C, 45 s at 56°C, and 45 s at 72°C, followed by eight cycles of 30 s at 94°C, 45 s at 53°C, and 45 s at 72°C, and then a final 10 min extension step at 72°C. Amplified fragments were analyzed using an ABI 3730XL (Applied Biosystems, USA) and genotypes were determined using the Genemarker program (version 1.85; Softgenetics LLC). To confirm whether selected fragments were correctly amplified, PCR for Sanger sequencing was performed using a slight modification of the conditions above: 16 pmol of forward and reverse primers were used, with the exception of fluorescently labeled M13. The amplified products were sequenced using an ABI 3730XL (Applied Biosystems, USA) and checked whether targeted sequences were correctly amplified.

### Statistical analysis for genotyping

Allelic variation at the microsatellite loci in the 20 individuals were determined as number of alleles per locus and heterozygosity. Heterozygosity and allelic frequencies were calculated using ARLEQUIN version 3.1[30]. The test for departures from Hardy-Weinberg equilibrium (HWE) comparisons were made between observed heterozygosity (Ho), and expected heterozygosity (He) using exact tests as implemented by ARLEQUIN. Tests for null alleles were performed with MICRO-CHECKER version 2.2.3 [31].

### Comparison of genotyping efficiency

To compare the experimental efficiency between the traditional and NGS methods, we applied the seven markers reported by Hu and co-workers [25] to the Korean water deer population. These microsatellite markers were originally developed from cattle (*B. taurus*) and applied to Chinese water deer. PCR amplification and loci verification was performed as described previously. To compare genotype sizes, 1 bp was reduced from the original fragment size because the M13 sequence tag in this study was 1 bp smaller than that used by Hu and co-workers.

## Results

### Distribution of di- and tri-nucleotide repeats

**Figure 1:**
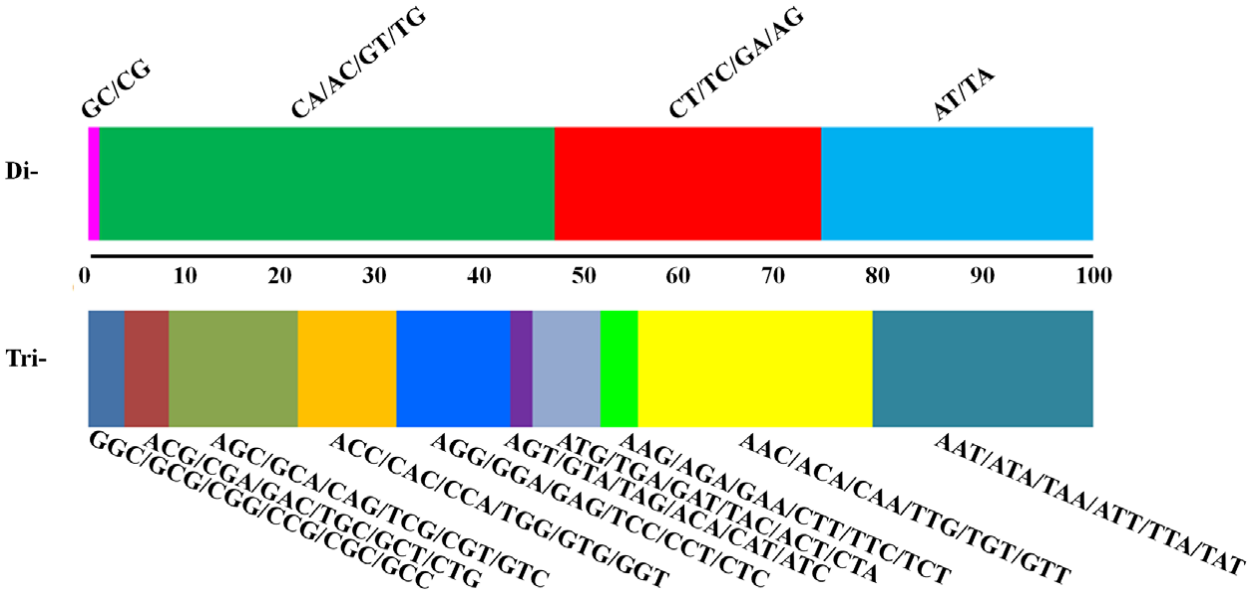
Frequency distribution of di- and tri-nucleotide repeats in motif sequences from Korean water deer.

Only perfect di- and tri-nucleotide microsatellites with a minimum four repeat copies in the 98-Mbp sequence were analyzed. A total of 20,101 regions containing perfect di-/tri-nucleotide repeats (204 repeats per million base pairs) were identified (Table 1). The observed frequency of different di- and tri-nucleotide types is shown in Fig. 1. Di-repeats were 5.9 times more common than tri-repeats (Table 1). The most frequent di-nucleotide was [CA]_n_ (79.72/Mbp), followed by [TA]_n_ (47.51/Mbp), [CT]_n_ (46.51/Mbp), and [GC]_n_ (1.94/Mbp). Of the tri-nucleotides, [AAC]_n_ and [AAT]_n_ were the most frequent (6.89 and 6.48/Mbp, respectively). The [AGT]_n_ repeat was the least common (0.63/Mbp). The difference between the most and least frequent repeats was 41- and 11-fold for di- and tri-repeats, respectively.

**Table 1:** Total occurrence of repeats in Korean water deer genomes and the number of primers designed.

Repeat types	No. of repeating units																								Total	Primers designed
	4	5	6	7	8	9	10	11	12	13	14	15	16	17	18	19	20	21	22	23	24	25	26	27		
**Total**	**14036**	**2925**	**1190**	**637**	**341**	**176**	**148**	**110**	**82**	**72**	**68**	**67**	**44**	**46**	**38**	**40**	**28**	**18**	**17**	**8**	**5**	**2**	**1**	**2**	**20101**	**14738**
**Di-nucleotide**	**11919**	**2446**	**1002**	**575**	**310**	**168**	**144**	**105**	**81**	**72**	**68**	**67**	**44**	**46**	**38**	**40**	**28**	**18**	**17**	**8**	**5**	**2**	**1**	**2**	**17206**	**12471**
AT/TA	3390	577	238	133	83	45	35	23	19	18	9	15	7	9	13	12	10	8	3	3	3	1	-	2	4656	3144
CT/TC/GA/AG	3688	527	159	80	46	21	7	5	1	4	1	-	5	1	-	-	-	-	2	-	-	-	-	-	4547	3605
CA/AC/GT/TG	4698	1,314	594	358	178	101	102	77	61	50	58	52	32	36	25	28	18	10	12	5	2	1	1	-	7813	5601
GC/CG	143	28	11	4	3	1	-	-	-	-	-	-	-	-	-	-	-	-	-	-	-	-	-	-	190	121
**Tri-nucleotide**	**2117**	**479**	**188**	**62**	**31**	**8**	**4**	**5**	**1**	**-**	**-**	**-**	**-**	**-**	**-**	**-**	**-**	**-**	**-**	**-**	**-**	**-**	**-**	**-**	**2895**	**2267**
AAT/ATA/TAA/TTA/TAT/ATT	476	106	27	12	4	3	3	3	1	-	-	-	-	-	-	-	-	-	-	-	-	-	-	-	635	498
AAC/ACA/CAA/TTG/TGT/GTT	494	105	43	17	11	2	1	2	-	-	-	-	-	-	-	-	-	-	-	-	-	-	-	-	675	544
ATG/TGA//GAT/TAC/ACT/CTA	90	6	5	5	1	-	-	-	-	-	-	-	-	-	-	-	-	-	-	-	-	-	-	-	107	78
AAG/AGA/TTC/TTC/TCT/CTT	160	21	16	1	-	-	-	-	-	-	-	-	-	-	-	-	-	-	-	-	-	-	-	-	198	152
AGT/GTA/TAG/TCA/CAT/ATC	45	11	4	-	-	2	-	-	-	-	-	-	-	-	-	-	-	-	-	-	-	-	-	-	62	42
AGG/GAG/GGA/TCC/CTC/TCC	263	48	12	3	1	1	-	-	-	-	-	-	-	-	-	-	-	-	-	-	-	-	-	-	328	260
ACC/CAC/CCA/TGG/GTG/GGA	226	45	11	2	2	-	-	-	-	-	-	-	-	-	-	-	-	-	-	-	-	-	-	-	286	241
AGC/CAG/GCA/TCG/CGT/GTC	213	97	42	15	4	-	-	-	-	-	-	-	-	-	-	-	-	-	-	-	-	-	-	-	371	284
ACG/CGA/GAC/TGC/GCT/CTG	83	19	18	3	3	-	-	-	-	-	-	-	-	-	-	-	-	-	-	-	-	-	-	-	126	97
GGC/GCG/CGG/CCG/CGC/GCC	67	21	10	4	5	-	-	-	-	-	-	-	-	-	-	-	-	-	-	-	-	-	-	-	107	71
**Ratio of Di- to Tri- nucleotides**	5.6	5.1	5.3	9.3	10.0	21.0	36.0	21.0	81.0																5.9	5.5

### Copy number and length of di- and tri-nucleotide repeats

The copy number of repeat units in di-repeats was twofold higher than in tri-repeats. All tri-repeat motifs showed a narrow distribution range below 12 copies and a lower frequency than di-repeats (Table 1). Di-repeats of up to 22 repeat units were detected at a high frequency; those of 23 units or above were at a low frequency, with the exception of [GC]_n_ (Table 1). Di-repeat motifs were longer than tri-repeats. The 27 repeat unit numbers (54 bp) were the greatest in di-repeat motifs; 12 repeat units (36 bp) were the greatest in tri-repeat motifs.

### Amplification of microsatellite markers

To examine the amplification of detected repeats, 12,471 and 2,267 primer pairs for di- and tri-nucleotide repeats, respectively, were designed. Allelic diversity of microsatellites is positively correlated with repeat length, and dimer repeats have been characterized as having higher mutation rates [11],[12]. Thus, we chose 400 microsatellites with higher copy numbers from all four di-repeat motifs for amplification and assessment of polymorphism (Table 2). Of these, 106 were successfully amplified with unambiguous alleles. The 79 markers were polymorphic with 2 to 11 alleles (total 334 alleles) (Table 2 and Table S1). Nucleotide sequences of the 79 novel microsatellite markers were deposited in GenBank under accession numbers HQ876092–HQ876170. Rates of polymorphism were 21% for [CA]_n_, 34% [CT]_n_, 19% [TA]_n_, and 5% [GC]_n_ (Table 2). Of these, TA repeats showed the highest allelic richness, an almost twofold higher number of alleles per locus than the other repeats (Table 2).

**Table 2:** Genotyping of newly designed di-nucleotide species-specific microsatellites and cross-species (cattle to water deer) microsatellites on Korean water deer (*n* = 20 individuals).

Types	No. of polymorphic markers	No. of alleles	No. of alleles per locus	Range of *H_e_*	Range of *H_o_*	Reference
**Species-Specific**	**79**	**334**	**4.23**			This study
AT/TA	21	137	6.52	0.05–0.78	0.00–1.00	
CA/AC/GT/TG	34	107	3.15	0.27–0.88	0.15–0.95	
CT/TC/GA/AG	19	72	3.79	0.05–0.77	0.00–0.80	
GC/CG	5	18	3.60	0.05–0.80	0.05–0.80	
**Cross-specific**	**3**	**14**	**4.67**			Hu et al., 2007
BM888	1	6	6	0.23	0.30	
BM3628	1	4	4	0.71	0.45	
TGLA10	1	4	4	0.54	0.40	

**Figure 2:**
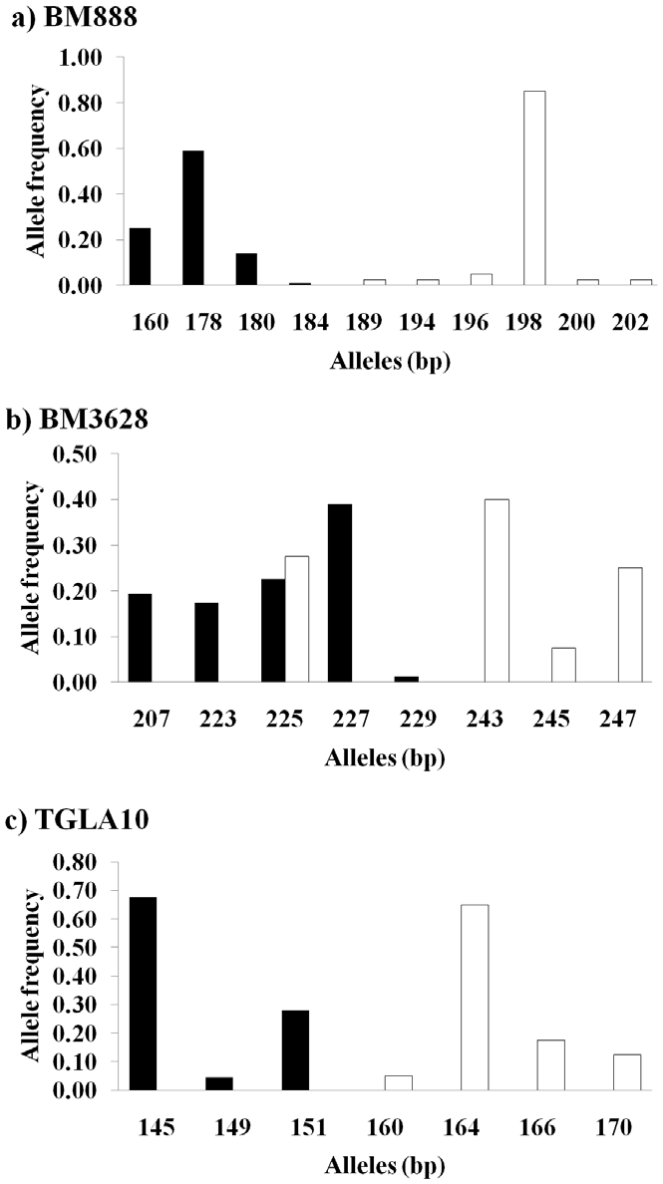
Microsatellite allele distributions in Chinese and Korean water deer populations. The cross-specific microsatellites were originally developed from cattle [25]. Black bars incidate frequency of allele size in Chinese water deer population and white bars in Korean water deer population. (a) BM888 locus (b) BM3628 locus (c) TGLA10 locus.

Among the seven microsatellites reported by Hu and co-workers [25], we were able to obtain the sequences of primers for six markers and applied these to the Korean water deer population. However, two primer sets did not result in successful amplification, and one amplified a different locus that had no repeats. Therefore, only three microsatellites were successfully amplified. A total of 14 alleles were detected in the 20 individuals examined; 6 at BM888, 4 at BM3826, and 4 at TGLA10 (Table 2; Fig. 2).

### Verification of perfect microsatellite markers for genotyping

The number of alleles, observed heterozygosity (*H_o_*), and expected heterozygosity (*H_e_*) for the 79 markers are summarized in Table 2. Analysis of the 79 new markers and 3 existing primer sets [25] did not show any evidence of significant linkage disequilibrium among loci in the Korean water deer population (Table 2 and Table S1). Departures from Hardy-Weinberg equilibrium (HWE) (*P*<0.05) were distributed across loci (Table S1), suggesting the possibility of null alleles at these loci, as determined with the MICRO-CHECKER software package. The majority of microsatellite markers exhibited a high rate of variation.

## Discussion

**Figure 3:**
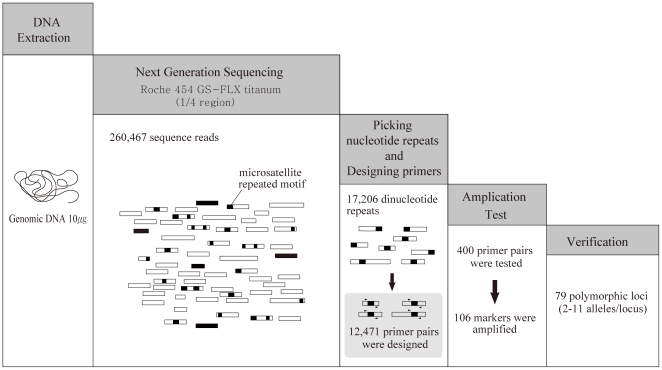
Diagrammatical representation of the NGS method of microsatellite isolation.

Nuclear microsatellites are one of the most popular types of molecular marker for population genetics. However, the use of microsatellites for studying populations of endangered/non-model species has been impeded by a lack of available sequence resources. The present study was designed to screen species-specific microsatellites for Korean water deer. Compared to the simple hybrid capture method, which is popular for developing microsatellites from species with little genetic information, the NGS method is fast, simple, and eliminates a number of technical difficulties. This method is composed of only five steps: i) isolation of genomic DNA, ii) NGS sequencing, iii) selection of nucleotide repeats and primers design, iv) amplification, and v) verification of amplification accuracy by direct sequencing (Fig. 3). Moreover, while traditional microsatellite development requires different libraries and probes for each type of repeat, the NGS method efficiently detects all kinds of repeats through the use of certain bioinformatics tools. In addition, the NGS method does not need high-depth coverage of the genome and thus a minimum run on the NGS platform is sufficient. Accuracy can be verified by PCR and direct sequencing to confirm the presence of that sequence and target repeats. Ideally, if 16 samples are loaded on one NGS run (two large regions), sufficient microsatellites for a population study are obtained at a minimum cost.

Di-nucleotide repeat motifs are usually more common than tri-repeats in eukaryotes such as fungi, *Drosophila*, *Caenorhabditis elegans*, and plants [32]–[34]. However, tri-repeats are more frequently detected in coding regions [35]–[38]. Because the insertion or deletion of one repeat copy of a tri-repeat motif does not generate a frame-shift mutation, such changes may be less evolutionarily constrained than changes in di-repeats and thus explain the higher frequency of tri-nucleotide repeats in coding regions. In the swordtail cricket, tri- and di-repeats were more common in coding and non-coding regions, respectively, than would be expected [11]. However, because coding regions occupy but a small portion of the genome (1.21% in the bovine genome), we expect that most repeats in this study reside in the non-coding region. Therefore, di-repeats should be significantly more common than tri-repeats. Assuming this is so for Korean water deer, we expect that tri-repeats are under relatively high selective pressure to avoid destruction of protein. Thus, expansion of repeats may be limited by functional constraints and may widen the gap between the numbers of dimer and trimer repeats at higher repeat numbers: 5.6 at 4 repeating units to 81.0 at 12 repeating units (Table 1). The appearance frequency of different types of repeats varies according to species [32]. For example, [CA]_n_ repeats are most frequent in humans, *Drosophila*, and water deer; [TA]_n_ repeats are so in crickets, *Arabidopsis*, and yeasts; and [CT]_n_ are so in mosses and *C. elegans*. However, the least common di-nucleotide repeat is most often [GC]_n_ in a variety of species, including water deer [11],[39]. Of the tri-nucleotide repeats, [AAT]_n_ and [AAC]_n_ are the most common in both water deer and humans [32].

Four hundred new microsatellite markers from Korean water deer were genotyped and 79 (20%) among them were successfully amplified with polymorphism. All microsatellite markers showed high diversity and variation, even among the 20 individuals tested. These data have significantly increased the resources available for the study of *H. inermis* genetic diversity and population structure. In a previous study on hog deer, of the 120 primer sets from 161 colonies positive for AC and AGC repeat motifs, only 9 (8%) resulted in successful amplification of two or three alleles from 26 individuals [40]. In a study on Pére David's deer (*Elaphurus davidianus*), only ∼10% (8/71) of the primer sets from 300 positive colonies resulted in successful amplification of two alleles in 58 individuals [41]. In another study that considered traditional microsatellite genotyping methods using cross-species markers, only three of seven primer sets resulted in successful amplification [25]. Taking into account the cross-species transferability from cattle to Chinese water deer and from Chinese to Korean water deer (i.e., 3/7 success ratio), NGS is an effective method for the development of species-specific polymorphic microsatellite markers.

The 79 new microsatellite markers detected in Korean water deer were highly variable; however, departures from HWE were evenly distributed among loci (*P*<0.05; Table 2 and Table S1). These data suggest a possible Wahlund effect, because we pooled samples from the whole of South Korea [42]. Therefore, this finding may be the result of admixture of more than two independent populations. Other possible reasons for the observed deviation from the Hardy–Weinberg equilibrium may be the presence of null alleles or a large allele dropout rate [43]. One of most important implications of this study is that the allele sizes of Chinese and Korean water deer gene pools were well-separated (i.e., larger genotypes in Korean water deer; Fig. 2). Because the genetic relationships between these subspecies remain debated [26] further studies using larger numbers of Korean water deer should be performed.

## Supporting Information

Table S1 Characteristics of 79 microsatellites isolated from Korean water deer (*n* = 20 individuals)
(DOCX)
