# Supplementary material for: Fast and Cost-Effective Mining of Microsatellite Markers Using NGS Technology: An Example of a Korean Water Deer Hydropotes inermis argyropus
Source: PLoS One. 2011 Nov 1;6(11):e26933. doi: 10.1371/journal.pone.0026933 (PMC3206051; doi:10.1371/journal.pone.0026933)
Supplement: Table S1 — Characteristics of 79 microsatellites isolated from Korean water deer (n = 20 individuals) (DOCX) [file pone.0026933.s001.docx]

**Table S1.** Characteristics of 79 microsatellites isolated from Korean water deer (n = 20 individuals)

| Locus | Repeats | Primer sequence (5'-3') | Size range (bp) | No. of Alleles | *H_o_* | *H_e_* | Accession number |
| --- | --- | --- | --- | --- | --- | --- | --- |
| HIca21_01 | (CA)_21_ | TGTAAAACGACGGCCAGTCTCGTTGGCTAATAAGCATTTC | 149-163 | 7 | 0.700 | 0.787* | HQ876092 |
|  |  | AAAACCAACAGTTCATCAGGTC |  |  |  |  |  |
| HIca21_02 | (CA)_21_ | TGTAAAACGACGGCCAGTAAAGAATTAGGCATGACTGAGC | 144-166 | 11 | 0.600 | 0.865* | HQ876093 |
|  |  | GACTGAGCACATACTTCCACAA |  |  |  |  |  |
| HIca20_01 | (CA)_20_ | TGTAAAACGACGGCCAGTCCTACTGTATAGCACAGGGGAA | 131-160 | 10 | 0.800 | 0.823 | HQ876094 |
|  |  | TAGTCATATATGCACACGCACA |  |  |  |  |  |
| HIca16_01 | (CA)_16_ | TGTAAAACGACGGCCAGTAAGATTCAGACAGGAAATGAGC | 110-124 | 6 | 0.450 | 0.754* | HQ876095 |
|  |  | ATCAGCACCAGAGTCTCATAGG |  |  |  |  |  |
| HIca16_02 | (CA)_16_ | TGTAAAACGACGGCCAGTTTTTAGACTGAGCTGTGTTCCA | 145-175 | 4 | 0.250 | 0.388* | HQ876096 |
|  |  | GCTCACACTAGAGCAGCGTAT |  |  |  |  |  |
| HIca15_01 | (CA)_15_ | TGTAAAACGACGGCCAGTATCCATTCATCCCAGGTTATAG | 144-174 | 7 | 0.800 | 0.782 | HQ876097 |
|  |  | CAGGTGGATCACACAGTAAAGA |  |  |  |  |  |
| HIca15_02 | (CA)_15_ | TGTAAAACGACGGCCAGTAGCATAAGGGGGAAAAAGTTAC | 116-142 | 10 | 0.650 | 0.853* | HQ876098 |
|  |  | CGGACACAACTGAAGTAGACTG |  |  |  |  |  |
| HIca15_03 | (CA)_15_ | TGTAAAACGACGGCCAGTATGGTAACCACAAAGCAAAAAC | 164-170 | 4 | 0.400 | 0.758* | HQ876099 |
|  |  | TTCTCTGTTACCTTCTTCTTCCC |  |  |  |  |  |
| HIca14_01 | (CA)_14_ | TGTAAAACGACGGCCAGTTGGCTTCTTTCAAGATTTCCT | 119-131 | 5 | 0.300 | 0.779* | HQ876100 |
|  |  | AACCTACAGAGAATCCAAAACG |  |  |  |  |  |
| HIca14_02 | (CA)_14_ | TGTAAAACGACGGCCAGTCAGGGGGCTACAGTTACACTAA | 175-179 | 3 | 0.350 | 0.524 | HQ876101 |
|  |  | ACTCCAGTATTCTTGCCTGAAA |  |  |  |  |  |
| HIca14_03 | (CA)_14_ | TGTAAAACGACGGCCAGTTAACCAGCCTTCCAAAACTAAA | 155-178 | 6 | 0.750 | 0.724 | HQ876102 |
|  |  | CAGTAATCTTGCCTGGAGAATC |  |  |  |  |  |
| HIca13_01 | (CA)_13_ | TGTAAAACGACGGCCAGTGTTCTATTGTCCAGAGAAGGCT | 154-160 | 4 | 0.700 | 0.688 | HQ876103 |
|  |  | GCAGAGAGTCAGACACAGCTTA |  |  |  |  |  |
| HIca13_02 | (CA)_13_ | TGTAAAACGACGGCCAGTAGCATTTGTGTGTTGTTGTTTT | 115-133 | 7 | 0.300 | 0.645* | HQ876104 |
|  |  | TGCCTTCTCAAGTAAGTTTTAGG |  |  |  |  |  |
| HIca12_02 | (CA)_12_ | TGTAAAACGACGGCCAGTGAGTCCAGATGCTGTGATGA | 146-158 | 7 | 0.750 | 0.792* | HQ876105 |
|  |  | TCTGGTTTCCTCTGACTTCTGT |  |  |  |  |  |
| HIca12_04 | (CA)_12_ | TGTAAAACGACGGCCAGTAGCTATCACAAAAATGCAACTG | 123-133 | 7 | 0.600 | 0.559 | HQ876106 |
|  |  | GGAGACGACTGAATGAATAAGC |  |  |  |  |  |
| HIca12_05 | (CA)_12_ | TGTAAAACGACGGCCAGTACTTTTCACTATTTTGCCAGGA | 117-129 | 4 | 0.450 | 0.499 | HQ876107 |
|  |  | TGTTCCCCCAATTAATTTTGTA |  |  |  |  |  |
| HIca12_06 | (CA)_12_ | TGTAAAACGACGGCCAGTCAGAGAAAACCACCACTGATCT | 131-143 | 6 | 0.300 | 0.767* | HQ876108 |
|  |  | TGATCTCATTACAACAAACAGCTC |  |  |  |  |  |
| HIca12_07 | (CA)_12_ | TGTAAAACGACGGCCAGTTCAGATCTGGAGGTTAAGCATT | 167-173 | 4 | 0.150 | 0.273* | HQ876109 |
|  |  | GCTCCATCATGGTCTACTGATT |  |  |  |  |  |
| HIca12_08 | (CA)_12_ | TGTAAAACGACGGCCAGTGAGTGAGAAAAGCAAAGCAGTC | 142-170 | 7 | 0.900 | 0.769 | HQ876110 |
|  |  | GTGTCCTGCTCAACTTCACTCT |  |  |  |  |  |
| HIca11_01 | (CA)_11_ | TGTAAAACGACGGCCAGTTCTCAGAAACTGTCACCA | 145-165 | 11 | 0.950 | 0.879 | HQ876111 |
|  |  | GCCATATATCCAAGACCATC |  |  |  |  |  |
| HIca11_02 | (CA)_11_ | TGTAAAACGACGGCCAGTCCTTTTCTGCATCAGCTT | 146-164 | 7 | 0.850 | 0.776 | HQ876112 |
|  |  | GATAGGCAGACAACAGGA |  |  |  |  |  |
| HIct11_01 | (CT)_11_ | TGTAAAACGACGGCCAGTGGGGAGAATTTAAGGTTCATGT | 123-142 | 9 | 0.800 | 0.758 | HQ876113 |
|  |  | GAGTCTGTCATGGAGAAAAAGG |  |  |  |  |  |
| HIct11_02 | (CT)_11_ | TGTAAAACGACGGCCAGTTTGGCAATATTCTCATTCTCCT | 175-189 | 6 | 0.600 | 0.744* | HQ876114 |
|  |  | CTGTGTCCATTAGGTAAAAGGG |  |  |  |  |  |
| HIct9_01 | (CT)_9_ | TGTAAAACGACGGCCAGTGCATGTTTAGAAATATGGCTCC | 139-141 | 2 | 0.050 | 0.050 | HQ876115 |
|  |  | TCCTTTCAATCCTTGGTATGTT |  |  |  |  |  |
| HIct9_02 | (CT)_9_ | TGTAAAACGACGGCCAGTCTCTGTGGCCATAGGAACTG | 101-169 | 6 | 0.500 | 0.601 | HQ876116 |
|  |  | AAATCAATTCTCAGGAGGGGT |  |  |  |  |  |
| HIct9_04 | (CT)_9_ | TGTAAAACGACGGCCAGTGTTTATGGACTGACAACGGACT | 117-165 | 5 | 0.500 | 0.426 | HQ876117 |
|  |  | GCTGGATGTTTTGTCTCAGATT |  |  |  |  |  |
| HIct9_05 | (CT)_9_ | TGTAAAACGACGGCCAGTGATACAATTAGGCAATGGAGGA | 164-168 | 3 | 0.700 | 0.569 | HQ876118 |
|  |  | TCCTGGTGTTCACCTTTAAAAT |  |  |  |  |  |
| HIct8_01 | (CT)_8_ | TGTAAAACGACGGCCAGTCTATAGCATCACAAAAATGGCA | 130-136 | 4 | 0.400 | 0.571 | HQ876119 |
|  |  | TGGAGAGTTGTTTTTCAAAAGG |  |  |  |  |  |
| HIct8_03 | (CT)_8_ | TGTAAAACGACGGCCAGTGCCTGTACAAATATACACAGGTTTT | 170-172 | 2 | 0.150 | 0.224 | HQ876120 |
|  |  | TGATGCTTGCATAACAACAGAT |  |  |  |  |  |
| HIct8_04 | (CT)_8_ | TGTAAAACGACGGCCAGTGAAATTGAGATCAAGAACGAGG | 152-158 | 2 | 0.150 | 0.142 | HQ876121 |
|  |  | CACTGGTTTCTTCTAGACAGGG |  |  |  |  |  |
| HIct8_05 | (CT)_8_ | TGTAAAACGACGGCCAGTAGCAATTGGACAGTTTTGAGAT | 170-172 | 2 | 0.500 | 0.508 | HQ876122 |
|  |  | AATGAGAGATGCCGCTAAACTA |  |  |  |  |  |
| HIct8_06 | (CT)_8_ | TGTAAAACGACGGCCAGTTTTCAACAAGTGTTACTGGACAA | 142-144 | 2 | 0.000 | 0.467* | HQ876123 |
|  |  | TCTGTGATCTGTTTTGATCAGC |  |  |  |  |  |
| HIct8_07 | (CT)_8_ | TGTAAAACGACGGCCAGTAGGACAAAACAAATCTTAGGCA | 124-134 | 4 | 0.600 | 0.654 | HQ876124 |
|  |  | TCAACAGAGAAGACAATGATGC |  |  |  |  |  |
| HIct8_07 | (CT)_8_ | TGTAAAACGACGGCCAGTTTCAAACCACTCCATTAACACA | 120-126 | 3 | 0.500 | 0.483 | HQ876125 |
|  |  | GTTGTGATAATTCCCTTCTGGA |  |  |  |  |  |
| HIct7_01 | (CT)_7_ | TGTAAAACGACGGCCAGTTGGATCCTGTTAAATTTGCTTT | 158-164 | 2 | 0.150 | 0.142 | HQ876126 |
|  |  | ATTCACTCCTCATATCCTCACG |  |  |  |  |  |
| HIct7_02 | (CT)_7_ | TGTAAAACGACGGCCAGTCTTTCAAGTGTACAATGGCGTA | 127-149 | 5 | 0.150 | 0.573* | HQ876127 |
|  |  | CCCTGCCTTCTTTCTCTTTACT |  |  |  |  |  |
| HIct7_03 | (CT)_7_ | TGTAAAACGACGGCCAGTATTCTGAGACCATGTCAGCAC | 121-127 | 3 | 0.650 | 0.514 | HQ876128 |
|  |  | CTGTACACAGTGAAGTTTGGGA |  |  |  |  |  |
| HIct7_04 | (CT)_7_ | TGTAAAACGACGGCCAGTATCTGCAGAATTAGTGGATGGT | 128-130 | 2 | 0.050 | 0.050 | HQ876129 |
|  |  | CTCTCTCCTGAGGACTCTGACT |  |  |  |  |  |
| HIct7_05 | (CT)_7_ | TGTAAAACGACGGCCAGTTTCCCTAACCATAGTTGGACTG | 158-162 | 3 | 0.200 | 0.190 | HQ876130 |
|  |  | GCTCATCATTTTGGTAGAAGGA |  |  |  |  |  |
| HIct7_06 | (CT)_7_ | TGTAAAACGACGGCCAGTCAAAACTCACATAGCAACACCA | 123-127 | 2 | 0.050 | 0.050 | HQ876131 |
|  |  | GAGCTTGTTTAGAGTCTCACGG |  |  |  |  |  |
| HIct7_08 | (CT)_7_ | TGTAAAACGACGGCCAGTTGAGTGAGCAGAGAGTAAGCTG | 176-182 | 3 | 0.700 | 0.554 | HQ876132 |
|  |  | ACAAATCCGCCAGGATTATAG |  |  |  |  |  |
| HIct7_09 | (CT)_7_ | TGTAAAACGACGGCCAGTGAAGACAGGGGAGAGTGTAGAG | 151-153 | 2 | 0.000 | 0.492* | HQ876133 |
|  |  | AACGAACAAGCAGAAAGAGAAG |  |  |  |  |  |
| HIct7_10 | (CT)_7_ | TGTAAAACGACGGCCAGTCAGATGGGCTAACATTCTCATT | 160-166 | 3 | 0.450 | 0.550* | HQ876134 |
|  |  | GTACTCTGCTATCTGGACTCCG |  |  |  |  |  |
| HIct7_10 | (CT)_7_ | TGTAAAACGACGGCCAGTTGAAGTGGCAAACTCTAAGTGA | 156-167 | 6 | 0.700 | 0.767 | HQ876135 |
|  |  | CAGCAGAAACTACACACAACATT |  |  |  |  |  |
| HIct7_11 | (CT)_7_ | TGTAAAACGACGGCCAGTGGAAAACCCAACAAAAACATAA | 123-125 | 2 | 0.100 | 0.185 | HQ876136 |
|  |  | ACTCGGGACTTAAGGCTCTCT |  |  |  |  |  |
| HIct6_02 | (CT)_6_ | TGTAAAACGACGGCCAGTTACCTGCAGACCTAGTCATGTG | 128-130 | 2 | 0.000 | 0.097* | HQ876137 |
|  |  | TTGGAAGTGTGGAGTCTTAACC |  |  |  |  |  |
| HIct6_04 | (CT)_6_ | TGTAAAACGACGGCCAGTGACTTGTGGTCTGGTGGGT | 157-163 | 2 | 0.100 | 0.097 | HQ876138 |
|  |  | GCATCACCCTGTCCTAGAGAT |  |  |  |  |  |
| HIct6_05 | (CT)_6_ | TGTAAAACGACGGCCAGTCACTGTGTCCTGACCTAGTGAA | 173-175 | 2 | 0.200 | 0.328 | HQ876139 |
|  |  | AGGTGATGGTATTTGGAGATTG |  |  |  |  |  |
| HIct6_07 | (CT)_6_ | TGTAAAACGACGGCCAGTGCATAGATACGTGTGTCTGCAT | 144-150 | 2 | 0.050 | 0.050 | HQ876140 |
|  |  | TTAATTTGGAGCCAAAGATGTC |  |  |  |  |  |
| HIct6_13 | (CT)_6_ | TGTAAAACGACGGCCAGTGCCTACTGTTTTCTACGGATGA | 137-161 | 3 | 0.150 | 0.145 | HQ876141 |
|  |  | CGTGCATGCATAGAATTAAAAA |  |  |  |  |  |
| HIct6_17 | (CT)_6_ | TGTAAAACGACGGCCAGTAGACACCAGCTCATTAGGTCTC | 136-140 | 2 | 0.150 | 0.142 | HQ876142 |
|  |  | TTCAGTCACTAAAATGAGGCAA |  |  |  |  |  |
| HIct6_18 | (CT)_6_ | TGTAAAACGACGGCCAGTCATGCTTGATATTTTTGGCATA | 155-171 | 4 | 0.400 | 0.696* | HQ876143 |
|  |  | TGAAGCTGATGTAAATGCACTC |  |  |  |  |  |
| HIct6_19 | (CT)_6_ | TGTAAAACGACGGCCAGTCCCCACTATAGCAACTTTTGTT | 159-169 | 2 | 0.050 | 0.050 | HQ876144 |
|  |  | TCTTGATAGAGTTGGGCAAGAT |  |  |  |  |  |
| HIct6_23 | (CT)_6_ | TGTAAAACGACGGCCAGTGACGAATACAATAAAAGGCCAG | 163-169 | 2 | 0.150 | 0.142 | HQ876145 |
|  |  | GTGACACTGATGACTGAAGTGG |  |  |  |  |  |
| HIct6_24 | (CT)_6_ | TGTAAAACGACGGCCAGTGAGGATACAACAAGGAGCAAAA | 136-142 | 3 | 0.250 | 0.368 | HQ876146 |
|  |  | GGTCACAGGTCGGATATCTCT |  |  |  |  |  |
| HIat10_01 | (AT)_10_ | TGTAAAACGACGGCCAGTTTCATTTAGTTTGTTTTCCAATTA | 159-177 | 4 | 0.750 | 0.663 | HQ876147 |
|  |  | AGTTAGGCAAAAATTACAAAAGTT |  |  |  |  |  |
| HIat9_01 | (AT)_9_ | TGTAAAACGACGGCCAGTTCACAAACGCTGAGGTAACATA | 145-149 | 3 | 0.500 | 0.477 | HQ876148 |
|  |  | CACTGGATATCAAAGAGTACAAAGAA |  |  |  |  |  |
| HIat9_02 | (AT)_9_ | TGTAAAACGACGGCCAGTCAGCATAATACAAGTGCCACTAA | 152-172 | 2 | 0.000 | 0.097* | HQ876149 |
|  |  | TTATGAATGACATGGAAATTCG |  |  |  |  |  |
| HIat8_01 | (AT)_8_ | TGTAAAACGACGGCCAGTCCCCTCAACTTTCACAGAAAT | 174-194 | 4 | 0.600 | 0.556 | HQ876150 |
|  |  | TTCTGTTATTAAAGCATAATGGAAAA |  |  |  |  |  |
| HIat8_02 | (AT)_8_ | TGTAAAACGACGGCCAGTAGTGTTCCATTTCAGAAGCATT | 123-127 | 3 | 0.500 | 0.594 | HQ876151 |
|  |  | ACACATGGCAAATTTTACATACA |  |  |  |  |  |
| HIat8_03 | (AT)_8_ | TGTAAAACGACGGCCAGTAGGGAATCAACAAGTTGTAAAAAG | 143-175 | 4 | 0.050 | 0.383* | HQ876152 |
|  |  | CACAACCACCATCACACATTAT |  |  |  |  |  |
| HIat8_04 | (AT)_8_ | TGTAAAACGACGGCCAGTAATATAACACGATTGTGGGTCA | 162-190 | 4 | 0.250 | 0.529* | HQ876153 |
|  |  | TCCTCTTTTCTTAACTTCTTGTAGTG |  |  |  |  |  |
| HIat8_05 | (AT)_8_ | TGTAAAACGACGGCCAGTAAAAATTAGAGTTTCTTGACTGAGAA | 153-163 | 4 | 0.550 | 0.585 | HQ876154 |
|  |  | TGTAGGCAGATGTCTTACCATC |  |  |  |  |  |
| HIat8_06 | (AT)_8_ | TGTAAAACGACGGCCAGTGCAGCTCATCATATTCTCCTTT | 139-147 | 3 | 0.150 | 0.312* | HQ876155 |
|  |  | TTATTCTGCTCCCTTCAATCAT |  |  |  |  |  |
| HIat8_07 | (AT)_8_ | TGTAAAACGACGGCCAGTAAAAATTAGAGTTTCTTGACTGAGAA | 134-140 | 3 | 0.450 | 0.542 | HQ876156 |
|  |  | CTGAGCTACCAGGAAATACCTT |  |  |  |  |  |
| HIat8_08 | (AT)_8_ | TGTAAAACGACGGCCAGTGTATAATTGAGGGCAAAGTCCA | 148-150 | 2 | 0.050 | 0.050 | HQ876157 |
|  |  | TAGGTTCAAATCCTCATTTGCT |  |  |  |  |  |
| HIat8_09 | (AT)_8_ | TGTAAAACGACGGCCAGTACATAAGAAAATCCAGCCTCCT | 158-174 | 5 | 0.600 | 0.610 | HQ876158 |
|  |  | AGTAACTGCCACTTGCTGAGTT |  |  |  |  |  |
| HIat8_10 | (AT)_8_ | TGTAAAACGACGGCCAGTGAGGAAAGGAAAGAAAGAAAAGA | 116-120 | 2 | 1.000 | 0.513* | HQ876159 |
|  |  | GTTCTTTCCCCTTGCAGTTAG |  |  |  |  |  |
| HIat8_11 | (AT)_8_ | TGTAAAACGACGGCCAGTAATGTAGCACCCATGGATACAG | 138-174 | 3 | 0.650 | 0.539* | HQ876160 |
|  |  | ATTTGCTAGGAAGGAGCATTTT |  |  |  |  |  |
| HIat7_01 | (AT)_7_ | TGTAAAACGACGGCCAGTTGCTTTTGCTATTATGGGTCTT | 118-170 | 8 | 0.550 | 0.779* | HQ876161 |
|  |  | TCAGCAAAAGTGTATCGTGTCT |  |  |  |  |  |
| HIat7_02 | (AT)_7_ | TGTAAAACGACGGCCAGTTCTTCCCAGTTAGTCCACAAGT | 164-198 | 8 | 0.150 | 0.780* | HQ876162 |
|  |  | CAGAGACAAACATTAGGTCTTGTAAA |  |  |  |  |  |
| HIat7_03 | (AT)_7_ | TGTAAAACGACGGCCAGTTGGGACACTTAATTCTCTCAGC | 239-255 | 5 | 0.450 | 0.775* | HQ876163 |
|  |  | AATTTTGTGTGGACAATTCACTT |  |  |  |  |  |
| HIat7_04 | (AT)_7_ | TGTAAAACGACGGCCAGTAAAATTAGTTGCGTTCATGGAG | 161-165 | 2 | 0.350 | 0.480* | HQ876164 |
|  |  | TTCAGATTTTCACATTTCATGG |  |  |  |  |  |
| HIat7_06 | (AT)_7_ | TGTAAAACGACGGCCAGTTCACCTATCTAACCTTTGCTTAAC | 169-177 | 3 | 0.400 | 0.415 | HQ876165 |
|  |  | GGAAAAGAATTTGAAAAAGAATG |  |  |  |  |  |
| HIgc6_01 | (GC)_6_ | TGTAAAACGACGGCCAGTCTTGGACGAGTTCGAGGAC | 136-140 | 2 | 0.050 | 0.050 | HQ876166 |
|  |  | ATAGTCGTCGCGGTATGGTA |  |  |  |  |  |
| HIgc4_02 | (GC)_4_ | TGTAAAACGACGGCCAGTGGGGTTTTTGTGTCGAATC | 163-175 | 3 | 0.100 | 0.190 | HQ876167 |
|  |  | GGAGCATGATGAAATCGC |  |  |  |  |  |
| HIgc4_03 | (GC)_4_ | TGTAAAACGACGGCCAGTTAGGTGTCGCGCCAGTTC | 144-160 | 7 | 0.800 | 0.800 | HQ876168 |
|  |  | ACGTACCCCTCACACCCAT |  |  |  |  |  |
| HIgc4_08 | (GC)_4_ | TGTAAAACGACGGCCAGTGATGAACCTCGCGCACTT | 162-168 | 4 | 0.150 | 0.146 | HQ876169 |
|  |  | GACGAACATCGACTCGAACA |  |  |  |  |  |
| HIgc4_09 | (GC)_4_ | TGTAAAACGACGGCCAGTGAAGACTCCCTGAGACCCC | 102-116 | 2 | 0.450 | 0.358 | HQ876170 |
|  |  | GTCCTGTTCCTACGCTTCG |  |  |  |  |  |

*H_e_*, expected heterozygosity; *H_o_*, observed heterozygosity; * indicates significant deviation from Hardy-Weinberg equilibrium (P < 0.05).
